# Diagnostic Accuracy of Charge-coupled Device Sensor and Photostimulable Phosphor Plate Receptor in the Detection of External Root Resorption In Vitro

**DOI:** 10.15171/joddd.2015.004

**Published:** 2015-03-04

**Authors:** Shirin Sakhdari, Zohreh Khalilak, Elham Najafi, Rezvaneh Cheraghi

**Affiliations:** ^1^Assistant Professor, Department of Oral & Maxillofacial Radiology, Maxillofacial Research Center,Islamic Azad University, Dental Branch, Tehran- Iran; ^2^Associate Professor, Department of Endodontics, Islamic Azad University, Dental Branch, Tehran- Iran; ^3^Post-graduate Student, Department of Endodontics, Faculty of Dentistry, Mashhad University of Medical Sciences, Mashhad, Iran; ^4^MS of Biostatistics, Department of Epidemiology and Reproductive Health at Reproductive Epidemiology Research Center, Royan Institute for Reproduc-tive Biomedicine, ACECR, Tehran, Iran

**Keywords:** Dental radiography, digital radiography, root resorption

## Abstract

***Background and aims.*** Early diagnosis of external root resorption is important for accurate treatment. The purpose of this study was to compare the efficacy of a charge-coupled device (CCD) sensor and a photostimulable phosphor (PSP) plate receptor in the diagnosis of artificial external root resorption.

***Materials and methods.*** In this diagnostic in-vitro study, 40 maxillary incisors were mounted in a segment of dry bone and preliminary radiographs were obtained using CCD and PSP sensors. Artificial resorption cavities were produced on the middle-third in half of the samples and on the cervical-third in the other half on the buccal root surfaces. Radiographs were repeated and images were evaluated. Data were statistically analyzed using chi-square and diagnostic tests.

***Results.*** There were no significant differences between the two sensors in the sensitivity (p=0.08 and 0.06) and specificity (p=0.13) for the diagnosis of resorption in both root areas. The overall accuracy of CCD was higher than PSP sensor; however, the difference was not statistically significance (p>0.05).

***Conclusion.*** CCD and PSP sensors chosen for the present study produced similar results in diagnosing simulated external root resorption.

## Introduction

External root resorption results in dissolution of cementum, dentin and sometimes extends toward the pulp. It has an unknown etiology in most cases; however, inflammation, tumors, and excessive mechanical and occlusal forces might contribute to the situation. One of the most common regions for external root resorption is the cervical area of the root, although it happens less probably in middle area too, and the highest incidence rate has been reported in anterior teeth.^1^ Root resorption was first described by Bell in 1830.^2^ In most cases, no clinical symptoms are present and it is usually detected during routine radiography. When such lesions are found, they are at an advanced level and treatment is almost impossible. In the early stages, diagnosis is based on the radiographic evidence of a resorption defect; therefore, detection of the lesion on a radiograph is very important for a dental practitioner. On the other hand, due to superimpositions, radiographs might fail to show lesions below certain diameters or depths.^3^

Recently, digital detectors have replaced radiographic films. Charge-coupled device (CCD) sensors have a silicon wafer as a base for image recording and the photo-stimulable phosphor (PSP) plates consist of polyester base, coated with a crystalline halide composed of europium-activated barium fluorohalide compounds.^4^ The quality of the image produced by a CCD detector depends on factors like chip pixel dimension, type of the scintillation layer, and acquisition and display software, while in PSP plates, the quality of the image may also depend on scanning procedure as well as electronics.^5^

Digital systems reduce radiation dose. They are time saving and offer image enhancement software, with easy communication and storage.^6^ Since the radiological diagnosis of external root resorption is important and considering the potential difference in diagnostic performance of these two digital detectors including their resolutions, this study was undertaken to compare the efficacy of these sensors in detecting external root resorption.

## Materials and Methods

**Figure 1.**
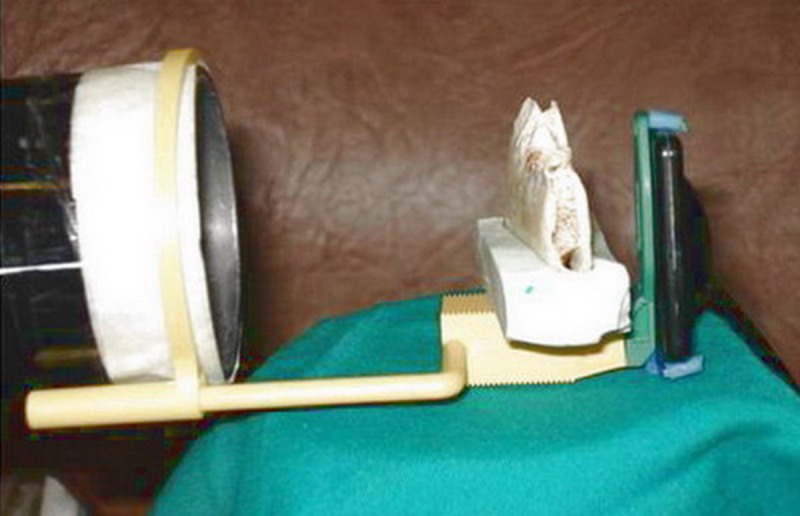
Dry alveolar block is fixed on a stand for an orthoradial radiograph.

In this diagnostic in-vitro study, 40 extracted maxillary incisors were selected. Teeth with root fractures, caries, external or internal root resorption, or anatomic variations on preliminary radiographs were excluded from the study. The teeth were immersed in 0.5% sodium hypochlorite solutions for 24 hours for disinfection. In order to simulate alveolar bone covering the tooth, a dry sheep alveolar block was provided and fixed on a firm flat stand perpendicular to the surface using a special jig. Teeth were numbered and consecutively placed within empty sockets. Radiographs were provided with both sensors under the same condition using a sensor holder (Kerr Sensor Holder, Hawa SA, Switzerland; Figure 1).

The focus-receptor distance was set at 20 cm and radiographs of the teeth were taken in an orthoradial direction. All images were exposed with a Minray radiographic machine (SOREDEX, Tusula, Finland) operated at 70kVp, 8 mA and exposure times of 0.03 and 0.12 seconds for PSP and CCD sensors, respectively, based on a pilot study for optimal image quality. Two digital sensors were used: Dr. Suni CCD detector (Suni Medical Imaging, San Joe, USA) size 2, offering 2 megapixels and pixel size of 22 μm with a resolution of 23 Lp/mm; and PSP detector (Digora^®^Optime; SOREDEX, Tusula, Finland) size 2 and scanner 40 μm (pixel), offering 12.5 Lp/mm spatial resolution. The exposed phosphor plates were immediately scanned.

The preliminary radiographs were saved in two separate files. Then the teeth were divided into two groups of 20 each. In group 1, artificial resorption defects (ISO 0.6 mm depth/0.6 mm diameter holes), were created on the buccal surfaces of the middle-third of the roots using a round 006 bur (Diatec, Switzerland).^2,3,7^ In group 2, the same procedures were carried out in the cervical-thirds of the roots. The size of defects were chosen based on the pilot study. The teeth were once again placed back to the sockets and radiographs were taken using the same sensors.

For each of the imaging systems, 80 radiographs (with and without resorption and with defects created in different locations) were obtained making a total of 160 radiographs. Before starting the observations, a precise definition of the radiographic appearance of the lesion was achieved among observers.

Images were coded and evaluated in a random order separately by two observers. Examiners evaluated each image without previous knowledge of the presence or location of the root surface cavities.

**Figure 2.**
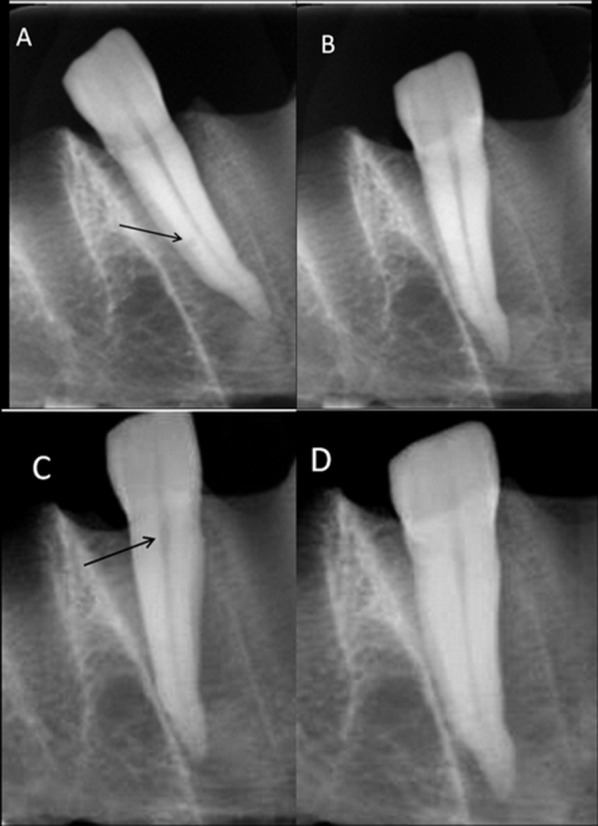
Example of images of premolar teeth. Arrows show the resorption cavities. A, B: Images of a tooth with and without cavity obtained by PSP sensor. C, D: Images of different teeth with and without cavity obtained by CCD sensor.

The results of the observations including the presence/absence and the location of resorptions were recorded for each tooth. The observation conditions were the same for all the images and they were displayed in a darkened room on a 15.6 inch laptop monitor (Dell, Inspiron N65, China) with a screen resolution set at 768×1366 pixels and color set to a 64-bit depth. Digital images were evaluated in their own software and the contrast and brightness tool of each system was used if necessary (Figure 2). Observations were made in 4 sessions with 2-week intervals. Diagnostic indices including the sensitivity, specificity, and accuracies of both sensors at different root areas, Kappa values for inter-observer agreement and Kappa values between sensors were calculated. Data were statistically analyzed using chi-square and diagnostic test at the significance level of 0.05.

## Results

The Kappa value (inter-observer agreement) between the observers for the CCD sensor was 89%. Also, the Kappa value between the observers for the PSP sensor was 90%.

A total of 160 digital images of teeth with and without resorption defects in the middle and cervical thirds with the CCD and PSP sensors were evaluated.

The observers of the CCD images found 15 (18.75%) of the total detected resorption defects to be in the middle thirds, 17 (21.25%) in the cervical thirds and none in the remaining 48 (60%), while 25% of them were actually found to have defects in the middle thirds, 25% in the cervical thirds and 50% remaining were found to have no actual resorption cavities.

The observers of the PSP images found 14 (17.5%) of the total detected resorption defects to be in the middle thirds, 19 (23.75%) in the cervical thirds and none in the remaining 47 (58.75%), while 25% of them were actually found to have defects in the middle thirds, 25% in the cervical thirds and 50% remaining were found to have no actual resorption cavities.

The diagnostic sensitivities in the middle-thirds, were 70% and 55% for CCD and PSP sensors, respectively; in the cervical-thirds, they were 75% and 60% for CCD and PSP sensors, respectively. There were no significant differences in the diagnostic sensitivities between the two sensors and the two root areas (P = 0.08 and P = 0.06, respectively).

The diagnostic specificities were 95% and 85% for CCD and PSP sensors, respectively. According to chi-square test, there were no significant differences in the diagnostic specificities between the two sensors (P = 0.13; Tables1 & 2).

**Table 1:** Distribution of readings (percent) with CCD sensor according to the presence or absence and location of the defect and standard method

	Gold standard			
CCD reading	Middle third	Cervical third	No defect	Total
Middle third	14(17.5%)	1(1.25%)	0	15(18.75%)
Cervical third	0	15(18.75%)	2(2.5%)	17(21.25%)
No defect	6(7.5%)	4(5%)	38(47.5%)	48(60%)
Total	20(25%)	20(25%)	40(50%)	80(100%)

**Table 2:** Distribution of readings (percent) with PSP sensor according to the presence or absence and location of the defect and standard method

	Gold standard			
PSP reading	Middle third	Cervical third	No defect	Total
Middle third	11(13.75%)	2(2.5%)	1(1.25%)	14(17.5%)
Cervical third	2(2.5%)	12(15%)	5(6.25%)	19(23.75%)
No defect	7(8.75%)	6(7.5%)	34(42.5%)	47(58.75%)
Total	20(25%)	20(25%)	40(50%)	80(100%)

The diagnostic accuracies were 83.75% and 71.25% with CCD and PSP sensors, respectively. This difference was not statistically significant (P > 0.05).

## Discussion

Dentists are faced with the challenge of timely diagnosis with regards to external root resorption, which can lead to a loss of tooth structure and a lower chance to preserve the tooth. Resorptive lesions are first diagnosed by intraoral radiographs, and therefore, the use of reliable radiographic techniques to diagnose such lesions is a necessity.^1,8,9^

In clinical situations, factors such as observation conditions, tissue superimposition and the position of the tooth involved in the dental arch influence the diagnosis of resorptive lesions. However, with in-vitro studies and elimination of confounding factors, the ability of detectors are studied more accurately.

In this study, teeth with artificial resorptive cavities were considered as the gold standard. The use of gold standard enabled the exact calculation of the percentage of positive and negative readings. Although borders of artificially created external root resorption cavities are relatively distinctive—and it seems that detecting such cavities are easier than that of natural cavities which have more diffuse borders, we could not employ a better method for creating artificial external root cavitations. In addition, bone marrow cavities imitated root defects, which possibly reduced the observers’ detection ability.

Since the results showed that the sensitivity and specificity for the diagnosis of the lesions were the same for both sensors in the two root segments studied, it seems that the observers’ ability to identify the defect was not affected by its location.

Although the CCD sensor revealed higher correct percentage of readings and the overall accuracy of CCD was higher than PSP sensor, differences were not statistically significant. Despite the higher resolution of the CCD sensor, the obtained results might have been due to software limitations.

Several studies have compared the digital systems and conventional films in detecting resorption defects;^2,3,7,10-12^ however, there are limited studies comparing CCD and PSP detectors in this respect. In a study by Kamburoğlu et al,^2^ conventional and digital radiography were compared in the diagnosis of external root resorption. Cavities were produced in the apical, middle and cervical-thirds on the two buccal and proximal root surfaces, which measured 0.5 and 0.8 mm in depth and cavities on the cervical and proximal areas were diagnosed much better.^2^ Also in a study by Dalili et al,^12^ cavities created using a bur in the middle-thirds were detected better than those in the cervical thirds, which is not in line with the findings of the present study.

Although in our study the cavities were created only on the buccal surfaces and only in the middle and cervical segments, the diagnostic efficacy of the lesions in the middle and cervical thirds was the same, which is similar to the results of the study by Kamburoğlu et al,^2^ where the CCD sensor performed better than the PSP sensor, probably due to low resolution of the PSP system.

In a study by Borg et al^10^ with the aim of comparing the efficacy of PSP and CCD sensors and conventional radiography in the diagnosis of external root resorption, both sensors yielded identical results, which is in accordance with our findings. In the latter study, cavities with 0.6 and 0.9 mm in diameter were created, and similar to the study carried out by Kamburoğlu et al,^2^ deeper lesions were diagnosed much better. Size is effective in detecting root defects particularly on intraoral radiographs.^7,10,13^ In the present study, all the lesions were 0.6 mm in depth, which is an appropriate size in the evaluation of the accuracy of the diagnosis of artificial resorption defects. In another study by Kamburoğlu et al^7^ carried out to diagnose internal resorption with the use of conventional radiography and various digital sensors, the results showed the number of correct diagnoses in the PSP images were less than other techniques, which is not in agreement with the results of the present study. In the latter study, the exposure times were 0.16 and 0.32 seconds with CCD and PSP sensors, respectively.^7^ PSP produces favorable images at higher x-ray doses, too, due to a higher dynamic range, which is considered a disadvantage for these systems.^14^

Given the disadvantages of conventional radiographs and the advances in digital radiography with the production of new sensors, the present study was undertaken to determine the superior digital system in the diagnosis of external root resorption.

Having similar sensitivities and specificities for both sensors in the diagnosis of resorptive lesions is clinically important. Generally, various factors can influence radiographic diagnoses. These include the imaging system (conventional vs. digital radiography), manipulation of images, observation conditions,^14,15^ and finally the type, dimension and location of the lesion. It appears that the use of technology including monitors, sensors, and appropriate software to assist in accurate diagnosis of various lesions can be very effective. However, another important consideration in addition to the familiarity of observers with hardware is their experience in working with digital images, especially operating the relevant computer programs. This has a significant role in the accuracy and correctness of the diagnosis.^16-20^Three-dimensional CBCT assessment has also been demonstrated for diagnosis and treatment planning of root resorption.^21-23^However, the use of digital intraoral sensors should be continued in this regard as doses of currently used CBCT units are still high.^24,25^

## Conclusion

Considering the methodology and the results of the present study, the chosen CCD and the PSP sensors produced similar results in diagnosing simulated external root resorption lesions.

## Acknowledgement

The authors would like to thank the Departments of Oral and Maxillofacial Radiology and Endodontics at Islamic Azad University of Tehran, Dental Branch for their support.
